# Plasma cytomegalovirus DNA load predicts outcomes in liver transplant recipients

**DOI:** 10.1002/iid3.371

**Published:** 2020-11-03

**Authors:** Hao‐Chien Hung, Po‐Jung Hsu, Jin‐Chiao Lee, Yu‐Chao Wang, Chih‐Hsien Cheng, Tsung‐Han Wu, Ting‐Jung Wu, Hong‐Shiue Chou, Kun‐Ming Chan, Wei‐Chen Lee, Chen‐Fang Lee

**Affiliations:** ^1^ Department of Liver and Transplantation Surgery Chang‐Gung Memorial Hospital at Linkou Taoyuan City Taiwan; ^2^ Department of General Surgery Chang‐Gung Memorial Hospital at Linkou Taoyuan City Taiwan; ^3^ Chang‐Gung University College of Medicine Taoyuan City Taiwan

**Keywords:** cytomegalovirus, liver transplant, outcome, real‐time qPCR, viral load

## Abstract

**Objective:**

Cytomegalovirus (CMV) infection has a significant negative impact on liver transplant (LT) recipients. We aimed to evaluate the efficacy of real‐time DNA quantitative polymerase chain reaction (qPCR) in the early detection of CMV and predicting post‐transplant outcomes.

**Materials and Methods:**

This was a retrospective study that enrolled a total of 49 adult LT recipients between December 2016 and October 2019. Serial CMV qPCR were tested weekly. We used operating characteristic curve analysis to quantify qPCR replication numbers to decide the optimal threshold to predict posttransplant complications and overall survival.

**Results:**

The optimal cut‐off value of 180 copies/ml (=164 IU/ml) was determined. We had 40 patients in the low qPCR group (<180 copies/ml) and nine patients in the high qPCR group (≥180 copies/ml). Higher qPCR was associated with more severe CMV disease, early allograft dysfunction, major posttransplant complications, longer ICU stays, and lower 2‐year overall survival (OS; all *p* < .05). In the univariate logistic regression model, persistent DNAemia ≥ 4 weeks after anti‐CMV treatment, coexisted bacterial and/or fungal infection, and high CMV qPCR ≥ 180 copies/ml with *p* < .100. High CMV qPCR ≥ 180 copies/ml (*p* = .016; hazard ratio [HR] = 19.5; 95% confidence interval [CI] = 1.73–219.49) remained to be the only independent risk factors for major complication by the multivariate analysis. The overall 2‐year OS rates were 92.5% and 66.7% in the low and the high qPCR group, respectively (*p* = .030).

**Conclusion:**

Our findings support evidence that qPCR is effective in detecting CMV infection provides an objective perspective in predicting posttransplant outcomes. High plasma CMV DNA load (defined as CMV qPCR ≥ 180 copies/ml or 164 IU/ml) not only indicates a hazard in developing major posttransplant complications but also associates with prolonged and refractory treatment courses.

## Graphical abstract

We described that high DNA replication (164 IU/ml) is an indicator of developing major postliver transplant complications. Implementing preemptive treatment for this high‐risk group is mandatory to improve outcomes after liver transplantation.

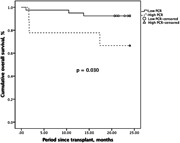

**Abbreviations**
ALT: alanine aminotransferase
AST: aspartate aminotransferase
AUROC: area under the receiver operating characteristic curve
CI: confidence interval
CMV: cytomegalovirus
GRWR: graft‐recipient weight ratio
HR: hazard ratio
ICU: intensive care unit
IU: International Units
LT: liver transplantation
MELD: model for end‐stage liver disease
OS: overall survival
qPCR: quantitative polymerase chain reaction

## 1 INTRODUCTION

Cytomegalovirus (CMV) is the most common single virus that causes complications in transplant recipients, and influences the outcomes and survival especially during the first few months after transplantation.^1^, ^2^, ^3^ Uncontrolled CMV infection in this particular immunosuppressed population could attribute to poor graft or patient outcome and even mortality after solid organ transplantation which brings up the issue of prophylaxis or preemptive antiviral therapy.^4^, ^5^ To identify CMV infection, multiple modalities are available including rapid shell vial culture technique, viral quantified methodologies such as CMV pp65 antigenemia assay, and real‐time CMV DNA quantitative polymerase chain reaction (qPCR).^6^

Current treatment strategies such as prophylaxis or pre‐emptive therapy for liver transplantation (LT) patients can be initiated and guided based on the detection of CMV.^7^ Early recognition with adequate intervention for CMV infection optimizes clinical outcomes after LT.^8^ It has been documented that the initial CMV viral loads and the viral loading increasing rate were associated with CMV disease after LT.^9^ Detection of high viral load before the occurrence of severe CMV disease could provide valuable messages for clinical treatment decisions.

Currently, CMV qPCR is the preferred and widely used CMV detection method in transplant recipients due to its high sensitivity, simplicity, and quickness.^10^, ^11^, ^12^ Yet, an ideal quantitative criterium for the prediction of critically severe postoperative outcome and diagnosis of severe CMV disease in CMV viral loads, has not been established for patients after LT. Besides, qPCR was designed for viral DNA detection, it may not be able to distinguish the difference between infected cell destruction and active viral replication.^13^ In the present study, we planned to validate the efficacy of CMV qPCR by comparing with the results of pp65 antigenemia assay. Furthermore, we aimed to disclose the association between CMV qPCR and LT outcome and provide a practical suggestion in anti‐CMV agents use according to an adequate cut‐off value of CMV qPCR.

## 2 MATERIALS AND METHODS

### 2.1 Study population

This study was a retrospective analysis of collected data. A total of 49 adult cases received LT in Chang Gung Memorial Hospital at LinKou, between December 2016 and October 2019, were tested for serial pp65 antigen assays and CMV qPCR for CMV detection during their hospitalization after transplant. All patients satisfied with a standard indication of LT for acute liver failure, end‐stage liver disease, or hepatocellular carcinoma. This study was approved by our Institutional Review Board (No. 202001326B0).

### 2.2 Liver transplant and clinical data collection

Surgical planning and execution were performed in consistency technique as our protocol, and it did not include a convention of splenectomy.^14^, ^15^ The details and results of our pretransplant preparation and posttransplant immunosuppressant use were explained clearly in our previous publication.^16^ Concerning posttransplant care, intensive care unit (ICU) was necessary. The length of stay depended on the process of getting stabilization. Documentation of associated clinical information and laboratory data were collected including recipient and donor age, gender, model for end‐stage liver disease (MELD) score, primary etiology of liver disease, type of LT, graft‐recipient weight ratio (GRWR), ventilator dependence days, length of ICU stays, hospitalized days, and CMV‐related data.

### 2.3 CMV serologic study

2.3.1

2.3.1.1

Before transplantation, the serologic tests that detect CMV antibodies (Immunoglobulin [Ig] M and IgG) in donors and recipients were measured by the enzyme‐linked immunosorbent assay. A positive CMV IgG indicates a previous CMV‐infected event. To act in concert with an IgG avidity test, it helped to distinguish a primary CMV infection (high binding strength) from a past episode (low binding strength) in IgM positive patients.

### 2.4 Standardizing CMV surveillance

To determine whether CMV reactivation was influential for posttransplant outcome, we routinely checked CMV antigenemia assay and CMV qPCR weekly since the surgery until the time the patient was discharged or dead.

### 2.5 CMV pp65 antigenemia and qPCR assays

Ethylenediaminetetraacetic acid‐treated whole blood samples were collected simultaneously for the antigen assay and the CMV qPCR study. The antigenemia assay was conducted within 6 h of blood sample collection, using the MonoFluo^TM^ Kit CMV code 52206 Immunofluorescence Assay (Bio‐Rad). Monoclonal antibodies identify CMV pp65 antigen and could be visualized with a fluorescent secondary antibody. A positive antigenemia was defined as at least one antigen‐positive cell per 500,000 peripheral blood leukocytes displaying characteristic fluorescence.

The CMV DNA was isolated and quantified using the COBAS® AmpliPrep/COBAS TaqMan® CMV Test (Roche Molecular Systems, Inc) according to the manufacturer's instructions. The lower limit of quantitation of test is 137 International Units (IU)/ml. The conversion factor between CMV DNA copies/ml and IU/ml is 0.91 IU/copy (i.e., 1 copies/ml = 0.91 IU/ml) in this assay. A viremia was defined as a positive qPCR, that is, detectable CMV DNA in the blood, even if calculated IU/ml were below the lower limit of quantitation of test.

### 2.6 Definitions of CMV DNAemia and CMV disease

CMV DNAemia was determined as the detectable CMV antigen and/or CMV DNA in the patient's blood in the absence of clinical symptom or sign.^17^ To diagnose a CMV disease needed to acknowledge the existence of documented CMV infection and clinical symptoms, including unexplained fever, thrombocytopenia (<150 × 10^3^/μl), leukopenia (<4,000/μl), and/or atypical lymphocytosis (>5%).^18^ We made an extension definition from previous research, severe form CMV disease was qualified when it involved two or more organ systems, deteriorated progressively, and eventually led to organs failure.^19^

### 2.7 Preemptive anti‐CMV treatment

The initiation of anti‐CMV treatment started once CMV DNAemia was detected by either intravenous ganciclovir 5 mg/kg twice per day, or oral valganciclovir 900 mg per day, and the treatment stopped when CMV DNAemia vanished.

### 2.8 Clinical outcomes measurement

Instead of routine liver graft biopsy, acute rejection after LT was defined and treated as our previously published article by an elevation of serum aspartate aminotransferase (AST) and alanine aminotransferase (ALT) two times of normal upper limits in our institution (AST: 34 IU/L, and ALT: 36 IU/L) or a rise greater than 30 IU/L in one day.^8^ Clavien–Dindo classification^20^ was utilized for LT‐related complication evaluation. A major posttransplant complication, the primary outcome of the present study, was defined as Clavien–Dindo class IV (life‐threatening single or multiorgan dysfunction) or class V (mortality, defined as death within 30 days after LT procedure or during the same admission, regardless of length of stays). Overall survival (OS), the secondary outcome, was calculated from the surgical day to the date of death or the last follow‐up.

### 2.9 Statistical analysis

Quantitative parameters were expressed by median values and mean values ±*SD*, and numbers with percentages were used for qualitative ones. The quantitative comparison between the two main CMV detect methods was checked using bivariate Pearson correlation. The optimal cut‐off value of DNA replication copy numbers in qPCR to predict major posttransplant complications was determined by the Youden index and its discriminative ability was tested by the area under the receiver operating characteristic curve (AUROC). To compare the categorized variables between the high and the low qPCR groups, Pearson's *χ* ^2^ test was utilized. In the univariate logistic regression analysis, factors with a *p* value less than .100 were considered potential ones and further entered into the multivariate model with backward selection. The OS difference was evaluated by the Kaplan–Meier method. Two‐tailed *p* value was considered to be statistically significant if it was less than .05. All analyses were calculated using SPSS Statistics version 24.0 (SPSS Incorporation).

## 3 RESULTS

### 3.1 Characteristics of the entire population

Demographic data were summarized in Table 1. A total of 49 patients received LT, of which 42 (85.7%) were male gender. The median value of the MELD score was 14 (the mean score was 16.3 ± 8.7). Most of the patients received living donor liver transplant (*n* = 47, 95.9%), and in the majority of cases underwent the surgery with right lobe grafts (*n* = 43, 91.5%) with a median GRWR of 0.90% (the mean value: 0.95 ± 0.21%). The leading etiology was hepatitis B virus infection (*n* = 24, 49.0%), followed by alcohol use (*n* = 21, 42.9%). Among all, 23 (46.9%) patients had hepatocellular carcinoma. With regarding to CMV serologic tests, there were 49 (100.0%) and 41 (83.7%) patients that had positive anti‐CMV IgG positive results in recipients and donors, respectively. In the present study, none of the enrolled patients had a positive IgM result. After the surgery, weekly tests for CMV antigen assay yielded positive results in 22 (44.9%) patients, while 29 (59.2%) patients had detectable CMV DNA (viremia) in their blood. The median viral copies in 1 ml sample were 150 (the mean copy numbers: 231.4 ± 432.6) through CMV qPCR studies. The median length of posttransplant ICU stay was 11 days (the mean: 14.1 ± 8.0 days), and the median hospitalization was 28 days (the mean: 37.3 ± 34.4 days). Four (8.3%) patients had encountered major post‐transplant complications. After a median followed time of 28.5 months (the mean follow‐up: 27.6 ± 9.9 months), six patients died.

**Table 1:** Demographic characteristics of 49 patients after LT

Factors	Median or number	Mean ± *SD*	Range
General information			
Recipient age (y)	54.5	53.0 ± 9.7	28.1–69.8
Recipient gender, male	42 (85.7%)		
Recipient CMV IgG, positive	49 (100.0%)		
Donor age (y)	31.1	32.6 ± 10.7	18.5–59.7
Donor gender, male	24 (49.0%)		
Donor CMV IgG, positive	41 (83.7%)		
MELD score	14	16.3 ± 8.7	8–40
HBV infection	24 (49.0%)		
HCV infection	12 (24.5%)		
Alcohol use	21 (42.9%)		
HCC	23 (46.9%)		
Ascites (ml)	900	3,238.4 ± 5,550.3	0–28,800
LDLT	47 (95.9%)		
Right lobe graft^a^	43 (91.5%)		
GRWR, %^a^	0.90	0.95 ± 0.21	0.61–1.52
PP65 antigenemia, positive	22 (44.9%)		
PP65, maximum/per 500,000 leukocytes	0.0	1.9 ± 4.0	0–23
CMV Viremia, positive	29 (59.2%)		
CMV qPCR, maximum copies/ml	150	231.4 ± 432.6	0–2,243
Clinical outcomes			
Follow‐up period (mo)	28.5	27.6 ± 9.9	1.0–42.9
Two‐year mortality, cases	6 (12.2%)		
Major post‐transplant complication	4 (8.2%)		
Ventilator dependence, days	1	3.4 ± 8.5	1–49
Posttransplant ICU stay, days	11	14.1 ± 8.0	6–49
Posttransplant hospitalization, days	28	37.3 ± 34.4	21–250

Abbreviations: CMV, cytomegalovirus; GRWR, graft recipient weight ratio; HBV, hepatitis B virus; HCC, hepatocellular carcinoma; HCV, hepatitis C virus; ICU, intense care unit; LDLT, living donor liver transplantation; MELD, model for end‐stage liver disease; qPCR, quantitative polymerase chain reaction; *SD*, standard deviation.

^a^ Only calculated from LDLT cases.

### 3.2 The correlation between the CMV antigenemia and qPCR assays

**Figure 1:**
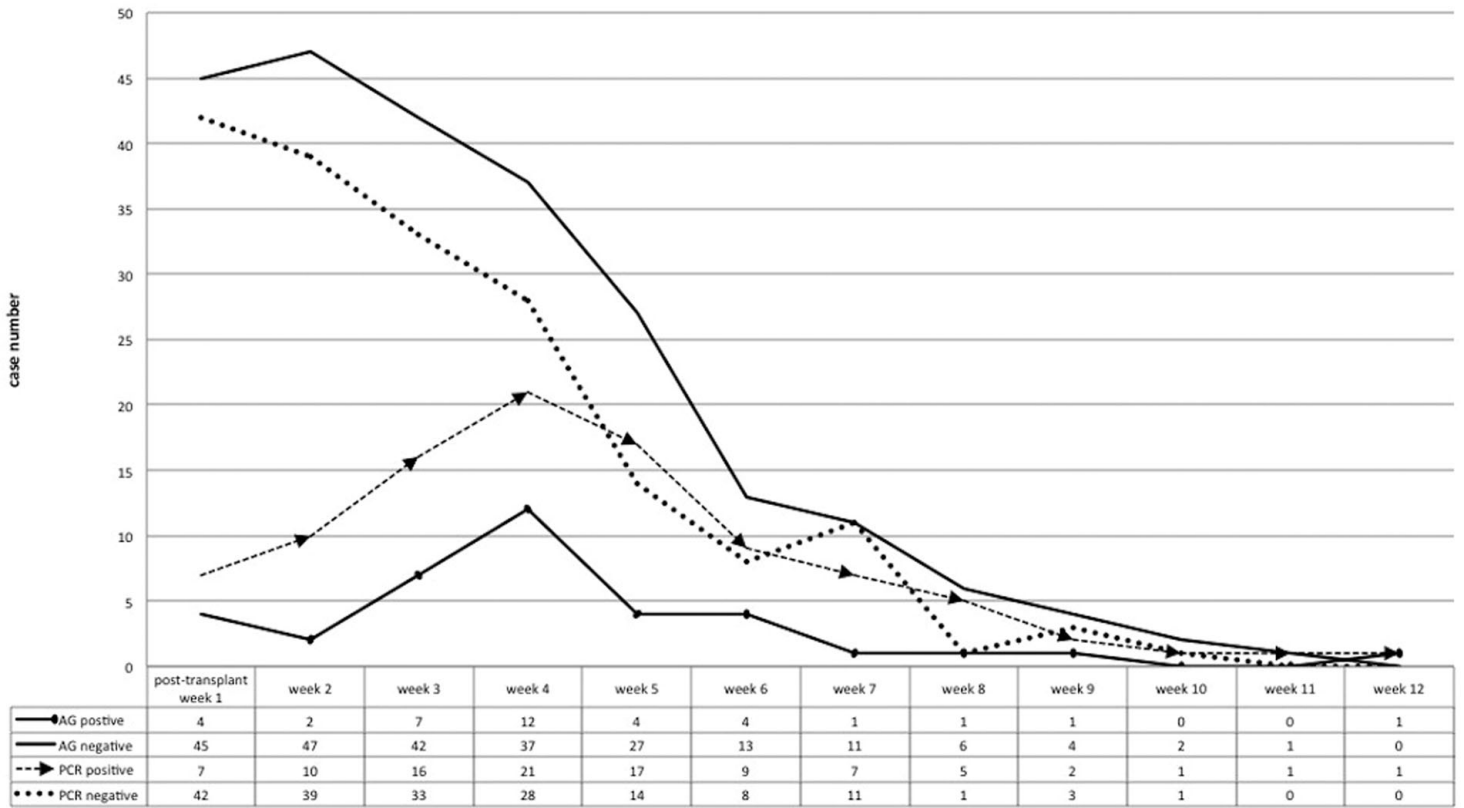
CMV pp65 antigenemia and CMV qPCR assays: qualitative results by week after LT. The CMV qPCR test demonstrated a better sensitivity in virus detection than the antigenemia assay. CMV, cytomegalovirus; LT, liver transplantation; qPCR, quantitative polymerase chain reaction

The quantitative correlation between the CMV antigenemia and qPCR assays was strong (*r* = .755, *p* < .001). The qualitative results between the two detection methods by week after transplant are shown in Figure 1. The CMV qPCR seemed to be detectable earlier than the antigenemia assay. In detail, all 29 patients with DNAemia, 20 (69.0%) of them had detectable viremia before a positive result came from the antigen assay.

### 3.3 Determination of the optimal cut‐off value of CMV qPCR

**Figure 2:**
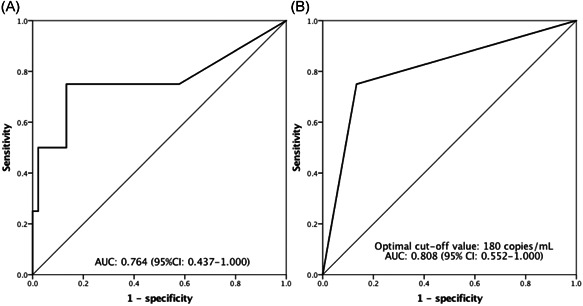
(A) ROC for the viral loads by the CMV qPCR affecting major complications after LT. (B) With an ideal cut‐off value of 180 copies/ml, the viral loads showed good discriminative power in predicting post‐LT major complications. CMV, cytomegalovirus; LT, liver transplantation; qPCR, quantitative polymerase chain reaction; ROC, receiver operating characteristic curve

In the first place, we used operating characteristic curve analysis to quantify qPCR replication copy numbers among individuals to decide the optimal threshold to predict the primary outcome (major posttransplant complication). The maximum copy number ever detected in each patient was used for this analysis. The AUROC of CMV qPCR copies in predicting major complication was 0.764 (95% CI: 0.437–1.000; Figure 2A). The optimal cut‐off value of 180 copies/ml (=164 IU/ml) was given by the Youden index, and the corresponded AUROC was 0.808 (95% CI: 0.552–1.000; Figure 2B). The correlated sensitivity and specificity values were 75.0% and 86.7%, respectively.

### 3.4 Comparison between the high and low qPCR groups

According to the optimal cut‐off value, we had 40 patients in the low qPCR group and nine patients in the high qPCR group. Under posttransplant weekly standardizing CMV surveillance, serial CMV qPCR data were gained for every individual patient. As long as the qPCR level ever exceeded the cut‐off value (180 copies/ml or 164 IU/ml), the patient would be assigned into the high qPCR group. Subsequently, we categorized 40 patients to the low qPCR group and 9 patients to the high qPCR group. Among the nine patients, five of them had persistent qPCR ≥ 180 copies/ml for 3 weeks and another four patients subsided after 1‐week treatment. A comparative study of posttransplanted patients according to high and low qPCR levels was further analyzed, as shown in Table 2. The proportion of older donor age (≥45 year old; *p* = .033) and higher MELD score (≥20; *p* = .029) were significantly higher in the high qPCR group. High qPCR levels were associated with detectable antigenemia (*p* = .028) and persistent DNAemia for 4 weeks after treatment (*p* < .001) compared to low levels. It exhibited no difference in early detectable DNAemia at postoperative week 1 between the two groups. Regarding clinical outcomes, higher qPCR seemed to be associated with more severe CMV disease, early allograft dysfunction, major posttransplant complication, longer ICU stays, and lower two‐year OS (all *p* < .05). Besides, a trend to have coexisted bacterial and/or fungal infection was observed in the high qPCR group (*p* = .072). However, the acute rejection rate distributed equally among the high and low qPCR groups (*p* = .243).

**Table 2:** Baseline demographics in LT patients according to low and high viral loads

Factors	Low qPCR, *n* = 40	High qPCR, *n* = 9	*p*
General information			
Recipient age (y, ≥60)	14 (35.0%)	2 (22.2%)	.460
Recipient gender (male)	35 (87.5%)	7 (77.8%)	.451
Donor age (y, ≥45)	3 (7.5%)	3 (33.3%)	**.033**
Donor gender (male)	17 (42.5%)	7 (77.8%)	.056
MELD score (≥20)	8 (20.0%)	5 (55.6%)	**.029**
HBV infection	18 (45.0%)	6 (66.7%)	.240
HCV infection	10 (25.0%)	2 (22.2%)	.861
Alcohol use	19 (47.8%)	2 (25.0%)	.219
HCC	20 (50.0%)	3 (33.3%)	.365
Ascites, mL (≥3,000)	11 (27.5%)	4 (44.4%)	.319
GRWR, % (<0.8)	11 (27.5%)	3 (42.9%)	.412
Donor CMV IgG, positive	33 (82.5%)	8 (88.9%)	.639
Detectable antigenemia	15 (37.5%)	7 (77.8%)	**.028**
Detectable DNAemia at POW 1	4 (10.0%)	3 (33.3%)	.438
Persistent DNAemia for 4 weeks	4 (10.0%)	7 (77.8%)	**<.001**
Clinical outcomes			
CMV infection status	20 (50%)	9 (100.0%)	**.006**
Asymptomatic (DNAemia only)	15 (20.0%)	3 (22.2%)	**<.001**
Disease: mild	5 (12.5%)	3 (33.3%)	
Disease: severe	0 (0.0%)	3 (33.3%)	
Acute rejection	10 (25.0%)	4 (44.4%)	.243
Early allograft dysfunction	7 (17.5%)	5 (55.6%)	**.016**
Coexisted bacterial infection	10 (25.0%)	5 (55.6%)	**.072**
Major postop complication ≥ Gr.IV	1 (2.5%)	3 (33.3%)	**.002**
ICU stay ≥ 21 days	2 (5.0%)	2 (22.2%)	**.008**
Two‐year mortality	3 (7.5%)	3 (33.3%)	**.033**

Abbreviations: CMV, cytomegalovirus; GRWR, graft recipient weight ratio; HBV, hepatitis B virus; HCC, hepatocellular carcinoma; HCV, hepatitis C virus; ICU, intense care unit; LT, liver transplantation; MELD, model for end‐stage liver disease; POW, postoperative week; qPCR, quantitative polymerase chain reaction.

### 3.5 Univariate and multivariate analysis logistic regression for major posttransplant complication prediction

Available clinical factors were analyzed in posttransplanted patients to predict major posttransplant complications, and the results are shown in Table 3. In the univariate logistic regression model, persistent DNAemia ≥ 4 weeks after anti‐CMV treatment, coexisted bacterial and/or fungal infection, and high CMV qPCR ≥ 180 copies/ml with *p* < .100. High CMV qPCR ≥ 180 copies/ml (*p* = .016; hazard ratio [HR] = 19.5; 95% confidence interval [CI] = 1.73–219.49) remained to be the only independent risk factors for major complication, as shown by the multivariate analysis.

**Table 3:** Uni‐/multivariate analyses of risk in predicting major post‐transplant complication

		Univariate			Multivariate	
	HR	95% CI	*p*	HR	95% CI	*p*
Persistent DNAemia ≥ 4 weeks	13.9	1.27–15 1.23	**.031**			
Coexisted other infection	8.25	0.78–87.17	.079			
High CMV qPCR ≥ 180 copies/ml	19.5	1.73–219.49	**.016**	19.5	1.73–219.49	**.016**

*Note*: Only significant results were listed.

Abbreviations: CI, confidence interval; CMV, cytomegalovirus; HR, hazard ratio; qPCR, quantitative polymerase chain reaction.

### 3.6 Comparison of OS rate according to high and low CMV qPCR copy numbers

**Figure 3:**
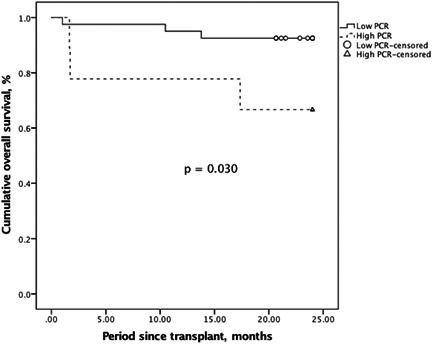
Kaplan–Meier method for overall survival comparison according to low and high CMV qPCR levels, and the latter indicated an inferior outcome. CMV, cytomegalovirus; PCR, polymerase chain reaction

After a median follow‐up period of 28.5 months, the overall 2‐year OS rates were 92.5% and 66.7% in the low and the high qPCR group, respectively. The recipients with higher levels of CMV qPCR demonstrated poorer survival outcomes compared to those who had low levels (*p* = .030; Figure 3).

## 4 DISCUSSION

High CMV replication has been considered to be associated with the risk of development of CMV disease and major complications after LT.^21^ In the present study, we defined high qPCR as viral loads over 180 copies/ml (164 UI/ml). The high qPCR group (≥180 copies/ml) was associated with higher incidences of developing severe form CMV disease, and it was also connected to a prolonged treatment course for greater than four weeks before CMV DNAemia became imperceptible.

Our data revealed a strong quantitative correlation between the two assays, CMV pp65 antigen, and qPCR. Although one research revealed that the quantitative antigenemia level seemed to be an equal growth of the corresponding viral loading measured by CMV qPCR.^11^ The result was difficult to get verified in the present study because of the common lack of high level of antigenemia and viral loading cases, which may be contributed from our early strategy in pre‐emptive treatment. The qualitative result between the pp65 antigen test and the CMV qPCR could be poorly correlated; however, higher viral loads did be associated with detectable antigenemia according to a previous study targeting on hematopoietic stem cell transplant recipients.^22^ In our study, we found that CMV was detectable earlier by real‐time CMV qPCR than antigen assay. In detail, the median time of the examined interval between the two different CMV tests was 1 week, and 1.4 ± 0.5 weeks in the average value, consistent with a previous study focusing on different kinds of patients including transplant ones.^23^ After treatment, the antigenemia assay achieved a negative result earlier than the qPCR with a median difference of 1 week (the average difference: 1.3 ± 1.5 weeks). Similar results were demonstrated by previous studies, which revealed a median difference from 9.5 to 10.5 days that the antigen assay became negative earlier than the qPCR did.^21^, ^23^

Without prevention, 18%–29% of all LT recipients may develop CMV disease.^24^, ^25^, ^26^ The incidences decrease to between 8% and 19%, among positive CMV serologic test recipients after LT.^24^ CMV syndrome accounts for the majority of CMV disease after LT, manifesting as fever and bone marrow suppression. Progression of CMV disease may clinical involve organ system, and cause irreversible dysfunction even death.^2^ As a potent upregulation of alloantigens, CMV not only increases the risk of acute rejection after transplant but also chronic allograft failure.^27^, ^28^ Besides, CMV infects the vascular endothelial cells, it is possible to lead to vascular and hepatic artery thrombosis.^29^ Furthermore, CMV possesses an immunomodulatory effect that may invite opportunistic bacterial, fungal, and other viral coinfections.^30^ The interaction between hepatitis C virus recurrence and CMV reactivation was also observed.^31^ On the other hand, bacterial and fungal infections may also be elements conditioning CMV disease after LT, so as old recipient age, female gender, high Charlson comorbidity index, diabetes mellitus, and high‐dosed mycophenolate mofetil.^32^, ^33^, ^34^ A prolongation of treatment course may also elicit incomplete recovery of CMV‐specific T‐cell responses, of which plays an important role in controlling persistent CMV infection.^35^

It is essential to identify the high‐risk ones and apply anti‐CMV agents to prevent CMV infection from becoming complicated CMV disease to reduce the risk of posttransplant death.^36^ It is reasonable to use intravenous ganciclovir for patients in a fact of being regarded as more severe or revealing signs of organ dysfunction. The expected incidence of ganciclovir‐resistant CMV is low (<1%) in LT recipients,^37^ which is considered to be associated with major adverse events, and only scanty of treatment options were available.^38^ CMV resistance should be thought out when there is an elevation in quantitative CMV detection, and high viral replication, CMV seronegative status of the recipient, overbearing immunosuppressant use, and insufficient dose of anti‐CMV agents could all be responsible for certain situations.^39^ Clinical relapse is more frequent in circumstances of sustainable DNAemia at the end of anti‐CMV therapy.^10^ Based on these, we would like to make a prudent proposal for preemptive therapy under recommended weekly CMV qPCR screening: for patients with viremia above a defined threshold of 180 copies/ml (164 IU/ml), intravenous ganciclovir should be applied in view of being in a hazardous condition of developing severe form disease. If patients have detectable viremia without exceeding the cut‐off value, oral valganciclovir takes precedence and continue the quantitative viral loads monitoring. The antiviral therapy should be continued until viral DNA is unable to be discovered in consecutive blood samples. During treatment, an elevation of DNA replication or a persistent viremia should attach importance to disease progression.

To avoid major posttransplant complications, trading off specificity for sensitivity might be valuable clinically. To this end, we did calculate the sensitivity/specificity with lower cut‐off values: according to the uppermost Youden index, defined by sensitivity + specificity − 1, the top three cut‐off values of CMV qPCR copy numbers were suggested to be 180, 165, followed by 154 copies/ml. However, the two following cut‐off values did not really increase the diagnostic sensitivity (remained as 75.0%), but slightly decreased in specificity: 84.4% and 82.2%, respectively. Therefore, 180 copies/ml represents the best discrimination ability of diagnostic decisions. In addition, a higher cut‐off value allows clinicians to have a more flexible space to choose anti‐CMV regimens.

The data presented in our study strongly implied that the direct association between high CMV viral loads and adverse posttransplant outcomes, but it had several limitations in addition to the study design (retrospective analysis in a single center). First, the small sample size increased the likelihood of a Type II error. Second, this study was conducted in a single tertiary medical center, and contradiction from protocol bias between other facilities and ours may arise. To this end, the availability of an international standard will improve interlaboratory test result agreement. Third, all recipients in the present study were with positive CMV serologic tests, so a direct effect on disease development should not be ignored. Fourth, this study focused on CMV detection during hospitalization and its impact on posttransplant outcomes, the occurrence of late‐onset CMV disease is out of scope. Therefore, larger and prospective research works are required to confirm our results.

In conclusion, our findings support evidence that qPCR is effective and reliable in diagnosing CMV infection and being an early signal to initiate pre‐emptive therapy. Besides, it also provides an objective perspective in predicting post‐transplant care outcomes. High plasma CMV DNA load (defined as CMV qPCR ≥ 180 copies/ml or 164 IU/ml) not only indicates a hazard in developing major posttransplant complications but also associates with prolonged and refractory treatment courses. Implementing preemptive treatment for high‐risk patients is mandatory to improve overall outcomes after LT.

## CONFLICT OF INTERESTS

The authors declare that there are no conflict of interests.

## AUTHOR CONTRIBUTIONS

Hao‐Chien Hung and Po‐Jung Hsu participated in the writing of the paper, research design, performance of the research, and data analysis. Jin‐Chiao Lee, Yu‐Chao Wang, Chih‐Hsien Cheng, Tsung‐Han Wu, Ting‐Jung Wu, Hong‐Shiue Chou, and Kun‐Ming Chan participated in research design and performance of the research. Wei‐Chen Lee and Chen‐Fang Lee participated in research design and and data analysis.

## Data Availability

The data that support the findings of this study are available from the corresponding author upon reasonable request.
